# Sensitive and Specific Detection of Lumpy Skin Disease Virus in Cattle by CRISPR-Cas12a Fluorescent Assay Coupled with Recombinase Polymerase Amplification

**DOI:** 10.3390/genes13050734

**Published:** 2022-04-22

**Authors:** Chuanwen Jiang, Dagang Tao, Yuanchen Geng, Hao Yang, Bingrong Xu, Yingyu Chen, Changmin Hu, Huanchun Chen, Shengsong Xie, Aizhen Guo

**Affiliations:** 1 The State Key Laboratory of Agricultural Microbiology, Huazhong Agricultural University, Wuhan 430070, China; jcw2021@webmail.hzau.edu.cn (C.J.); yuanchen@webmail.hzau.edu.cn (Y.G.); yanghao97@webmail.hzau.edu.cn (H.Y.); chenyingyu@mail.hzau.edu.cn (Y.C.); chenhch@mail.hzau.edu.cn (H.C.); 2 College of Veterinary Medicine, Huazhong Agricultural University, Wuhan 430070, China; dagangtao@foxmail.com (D.T.); hcm@mail.hzau.edu.cn (C.H.); 3 Key Laboratory of Agricultural Animal Genetics, Breeding and Reproduction of Ministry of Education & Key Lab of Swine Genetics and Breeding of Ministry of Agriculture and Rural Affairs, College of Animal Science and Technology, Huazhong Agricultural University, Wuhan 430070, China; xubingrongxu@163.com; 4 Hubei Hongshan Laboratory, Wuhan 430070, China; 5 Hubei International Scientific and Technological Cooperation Base of Veterinary Epidemiology, International Research Center for Animal Disease, Ministry of Science and Technology of the People’s Republic of China, Wuhan 430070, China; 6 Key Laboratory of Development of Veterinary Diagnostic Products, Ministry of China Agriculture and Rural Affairs, Wuhan 430070, China

**Keywords:** lumpy skin disease virus, recombinase polymerase amplification, CRISPR-Cas12a, nucleic acid detection

## Abstract

Lumpy skin disease (LSD) is a severe and highly infectious pox disease of cattle caused by the lumpy skin disease virus (LSDV). To facilitate early control of LSD, this study aimed to develop a new rapid on-site LSDV detection method using an *orf068* gene-based recombinase polymerase amplification assay (RPA) coupled with a CRISPR-Cas12a-based fluorescence assay (RPA-Cas12a-fluorescence assay). The results showed that the sensitivity of our RPA-Cas12a-fluorescence assay for detecting LSDV *orf068* gene reached 5 copies/μL with plasmid as a template, and 10^2^ TCID_50_/mL with viral genomic DNA as a template. No cross-reaction with other common bovine viruses was observed. Further, an on-site RPA-Cas12a-fluorescence assay of 40 clinical samples from cattle with or without LSD showed a diagnostic sensitivity of 96.3% (95% CI: 81.0–99.9%) and specificity of 92.31% (95% CI: 62.1–99.6%), which was close to those of the quantitative PCR assay. Therefore, our RPA-Cas12a-fluorescence assay has promising prospects in on-site rapid LSDV detection.

## 1. Introduction

The lumpy skin disease virus (LSDV) is a member of the Capripoxviruses (CaPVs) genus, belonging to the Poxviridae family [1]. The LSDV genome is a linear double-stranded DNA (dsDNA) molecule; the 151 kbp genome consists of a central coding region bound by identical 2.4 kbp-inverted terminal repeats and 156 putative genes [2]. The LSDV causes lumpy skin disease (LSD) in cattle, which is characterized by fever, enlarged lymph nodes, firm circumscribed nodules on the skin and mucosa, ulcerative lesions, and mortality of about 5% [3]. Furthermore, LSD can cause a reduction in the milk production of cows, the growth of calves and fattening cattle, and the fertility of bulls [4]. Considering the major economic impact on cattle, LSD was listed as a notifiable disease by the World Organization for Animal Health (OIE) and newly stipulated as a Class Ⅱ disease by Chinese law in February 2022 [5]. LSD once was confined to most African and Middle Eastern countries. In August 2019, an outbreak of LSD in cattle was first reported in the Yili region of Xinjiang, China [6]. In the following two years, it spread to more than 15 provinces in China. Meanwhile, it has been epidemic in several Asian countries and has caused serious economic losses. Therefore, early, rapid, and accurate detection of LSD is critical for controlling disease spread.

The traditional field diagnosis of the LSDV mainly depends on clinical signals. However, it is difficult to diagnose mild and subclinical diseases [7]. The virus neutralization test (VNT) is the only serological test validated by the OIE [8], but the VNT is very time- and labor-consuming. Other serological tests based on the whole virus or the antigens such as *orf095*, *P32*, and *orf103* proteins mainly include the Enzyme-linked immunosorbent assay (ELISA) [9,10] and immunofluorescence antibody test [11]. However, the cross-reaction between the LSDV and other poxviruses might affect the specificity of the above-mentioned serological tests [12,13] and some tests require expensive equipment. In addition, several nucleic acid-based methods were developed for detecting the LSDV, such as polymerase chain reaction (PCR) targeting the *p32* gene [10,14] and quantitative PCR targeting the *GPCR*, *orf068*, and *orf074* genes [1,15]. However, their application is limited due to expensive equipment, professional operation, standard laboratories, and a long response time. Loop-mediated isothermal amplification (LAMP) and recombinase polymerase amplification (RPA) possess numerous advantages such as rapidity, low cost, and user-friendliness, but cross-contamination limits their application [16].

As a severe infectious animal disease, the LSDV needs to be diagnosed by on-site, rapid, and accurate methods. Recently, a CRISPR/Cas-based nucleic-acid detection method with high sensitivity and specificity has been developed [17]. This method has been successfully applied to detect the African swine fever virus [18,19,20,21], SARS-CoV2 [22,23,24,25], *Mycobacterium tuberculosis* [26,27], *Staphylococcus aureus* [28], Cryptosporidium [29], Human papillomavirus [17], Zika virus, Dengue virus [30], and other viruses. However, there are no reports on the application of the CRISPR/Cas-based method in the detection of the LSDV.

In this study, we developed an RPA-Cas12a-fluorescence assay for the rapid on-site detection of LSDV. The *poly(A) polymerase small subunit* (*orf068*) gene, a highly conserved gene of the LSDV, was chosen as the target to develop the CRISPR/Cas-based detection method. Our method could facilitate the early and rapid control of LSD.

## 2. Materials and Methods

### 2.1. Primer Design and Polymerase Chain Reaction

The primers (Table S1) were designed using Oligo 7.0 (PREMIER Biosoft) based on all the LSDV strains’ *orf068* gene sequences available in GenBank (https://www.ncbi.nlm.nih.gov/genbank/, accessed on 17 May 2021). Meanwhile, the sequence of LSDV/Russia/Saratov/2017 (GenBank accession no: MH646674.1) was used as the reference. The partial sequence of the *orf068* gene (518 bp) was commercially synthesized (TSINGKE, Wuhan, China) and cloned into pUC57 to obtain the pUC57-*orf068* plasmid. Subsequently, 25 μL 2 × PCR Master Mix (Takara, Beijing, China), 2.5 μL primer sets (LSDV-0rf068-F/R), 19 μL ddH_2_O, and 1 μL amplification template containing the appropriate copy number of *orf068* gene were added into a PCR tube. PCR was performed in a BIOER TC-XP-D Cycler (Bioer Technology, Hangzhou, China) as follows: 40 cycles of denaturation at 98 °C for 10 s, annealing at 58 °C for 15 s, extension at 72 °C for 30 s, and followed by a final extension at 72 °C for 5 min. Then, 3 μL of PCR products with a length of 401 bp were subjected to 1% agarose gel electrophoresis, and the remaining PCR was kept for nuclease assay.

### 2.2. Preparation of CRISPR RNA (crRNAs) Targeting LSDV orf068

Six crRNAs targeting the LSDV *orf068* complementary regions were designed by CRISPR-offinder software (http://biootools.com/, accessed on 17 May 2021) (Table S2). The online ClustalW software (https://www.genome.jp/tools-bin/clustalw, accessed on 20 May 2021) was used for predicting the sequence conservativeness. Afterward, a forward primer containing a T7 promoter and a reverse primer containing different crRNA sequences were designed (Table S1). The PCR product was purified using a PCR purification kit (QIAGEN, Dusseldorf, Germany). With the purified PCR product as the DNA template, an in vitro transcription reaction was performed using a HiScribe™ T7 High Yield RNA Synthesis Kit (New England Biolabs, MA, USA), following the manufacturer’s instructions. The crRNAs were purified with a Monarch RNA Cleanup kit (New England Biolabs, MA, USA), quantified with a spectrophotometer (Denovix, Wilmington, NC, USA), and then kept at −80 °C until use.

### 2.3. PCR or Quantitative PCR (qPCR) Coupled with CRISPR-Cas12a Assay

The copy number of pUC57-*orf068* was calculated. The pUC57-*orf068* DNA was 10-fold serially diluted from 5 × 10^5^ to 5 × 10^−1^ copies/μL. PCR was performed on the series dilution template, and the PCR products were subjected to 1% agarose gel electrophoresis. Each Cas12a cleavage reaction system with a total volume of 17 μL contained 250 nM Cas12a (New England Biolabs, MA, USA), 500 nM crRNA, 1.5 µM JOE-dye single-stranded DNA-fluorophore-quencher (ssDNA-FQ) reporter, 3 μL of above PCR product, and 2 μL of NEBuffer 2.1. After the preparation of the reaction system, the reaction tubes were immediately transferred to an Applied Biosystems^®^ 7500 real-time PCR system (Applied Biosystems, CA, USA) at 37 °C with one reading cycle per 90 s. Reverse primers containing crRNA complementary sequences were used as ssDNA activators (Table S1). The results were displayed as amplification curves.

### 2.4. Recombinase Polymerase Amplification (RPA) Coupled with CRISPR-Cas12a Assay

The copy number of the pUC57-*orf068* was calculated and the plasmid DNA was 10-fold serially diluted. The RPA primers were designed using NCBI online software (https://www.ncbi.nlm.nih.gov/, accessed on 10 June 2021). The traditional qPCR and related primer pairs were used as a control [15,31]. RPA reaction was performed on the series dilution system. Briefly, 0.4 µM forward and 0.4 µM reverse primer, 8.65 μL ultrapure water, and 29.5 μL TwistAmp rehydration buffer (TwistDx, Cambridge, UK) were mixed and transferred to the tube containing one single pellet aliquot. Then, 2.5 µL TwistAmp magnesium acetate (280 mM) was added into the tube, briefly vortexed, and quickly centrifuged. An equal amount (about 10 µL) of the mixed RPA reaction solution was transferred into four PCR strip tubes. Then, 1 µL serially diluted plasmid was added to each tube [24], and the RPA reaction was performed in a water bath as follows: 37 °C for 40 min and 95 °C for 2 min. Afterward, 3 µL RPA product was subjected to 2% agarose gel electrophoresis, and 3 µL of the remaining RPA products were kept for the Cas12a nuclease assay. The RPA reaction was optimized under different reaction conditions, including volume, temperature, time, and primers.

Sensitivity to LSDV *orf068* gene detection was compared between the CRISPR-Cas12a cleavage assay and traditional qPCR. Each amplification template containing the appropriate copy number of the *orf068* gene was added to the master mix containing 10 µL 2 × AceQ^®^ qPCR SYBR Green Master Mix (Vazyme, Nanjing, China) and 4 µM qPCR primer (forward and reverse). The qPCR reaction was performed in the CFX96 Touch real-time PCR System (BIO-RAD, CA, USA) with 45 cycles of 95 °C for 10 s and 60 °C for 30 s. Amplification and cycle threshold (Ct) values were observed and calculated. The primers used for LAMP and qPCR are listed in Table S1.

### 2.5. LSDV Detection Sensitivity by RPA-Cas12a-Fluorescence Assay

The LSDV was prepared as follows. Briefly, the Verda Reno (vero) cells were infected with the virus at a multiplicity of infection (MOI) of 0.1. At 7 days post-infection (dpi), the virus was collected to detect tissue culture infective dose (TCID_50_). The LSDV was 10-fold serially diluted from 1 × 10^6^ to 1 × 10^1^ TCID_50_/mL. The DNA of serially diluted LSDV was extracted using the EasyPure Viral DNA/RNA kit (TransGen Biotech, Beijing, China). Then, the LSDV detection sensitivity by the CRISPR-Cas12a-RPA method was evaluated, as described in Section 2.4. Finally, the LSDV detection results were verified by various methods including blue light, UV light, gel imaging system, and multi-functional microplate reading. Meanwhile, the qPCR was performed on the serially diluted DNA templates, as described above.

### 2.6. LSDV Detection Specificity by RPA-Cas12a-Fluorescence Assay

The bovine DNA extracted from Madin-Darby bovine kidney (MDBK) cells and pUC57-*orf068* were mixed at the mass ratio of 1:1, and the mixture was used as the template to evaluate the effect of background DNA on RPA-Cas12a-fluorescence assay. The DNA samples IBRV, *Salmonella enterica Dublin*, *Mycoplasma bovis HB0801* (*M.b*), *Mannheimia haemolytica* (*M.h*) A1, and *Shiga toxin-producing Escherichia coli* (*STEC*) were kept in our lab. The RNA samples BRSV, BPIV-3, BVDV, BCoV, and BRV were extracted using an RNA extraction kit (Transgen, Beijing, China) from positive samples, and their complementary DNA (cDNA) was synthesized through reverse transcription (RT) using HiScript^®^ II Q Select RT SuperMix (Vazyme, Nanjing, China). The primer pairs were listed in Table S1. The specificity of RPA primers was evaluated by adding the above-mentioned DNA or cDNA to the RPA reaction. Every reaction contained 100 ng of the templates. The RPA products were verified by 1% agarose gel electrophoresis. Finally, the amplicons were validated by the RPA-Cas12a-fluorescence assay with the LSDV-specific crRNA.

### 2.7. Diagnosis of Lumpy Skin Disease by RPA-Cas12a-Fluorescence Assay

The 27 cattle were diagnosed as LSD cases and 13 cattle as LSD-free cases through viral test-based qPCR by the Diagnostic Center of Animal Epizootic Diseases, Huazhong Agricultural University, Wuhan, China. The DNA was extracted using an EasyPure Viral DNA/RNA kit (TransGen Biotech, Beijing, China), and 1 μL DNA from each sample was used as the template to perform qPCR and RPA-Cas12a-fluorescence assay. Then the results of these two methods were compared.

### 2.8. Statistical Analysis

Statistical analysis was performed using GraphPad Prism 8. The data were expressed as means ± SEM (standard errors of the mean) of three biological replicates for each sample. The *p* values were determined by using the unpaired Student’s t-test with two tails. * *p* < 0.05, ** *p* < 0.01, *** *p* < 0.001, and **** *p*  < 0.0001 represented the 4 levels of statistical significant difference, respectively.

## 3. Results

### 3.1. Establishment of Cas12a-Fluorescence Assay for Detection of LSDV

**Figure 1:**
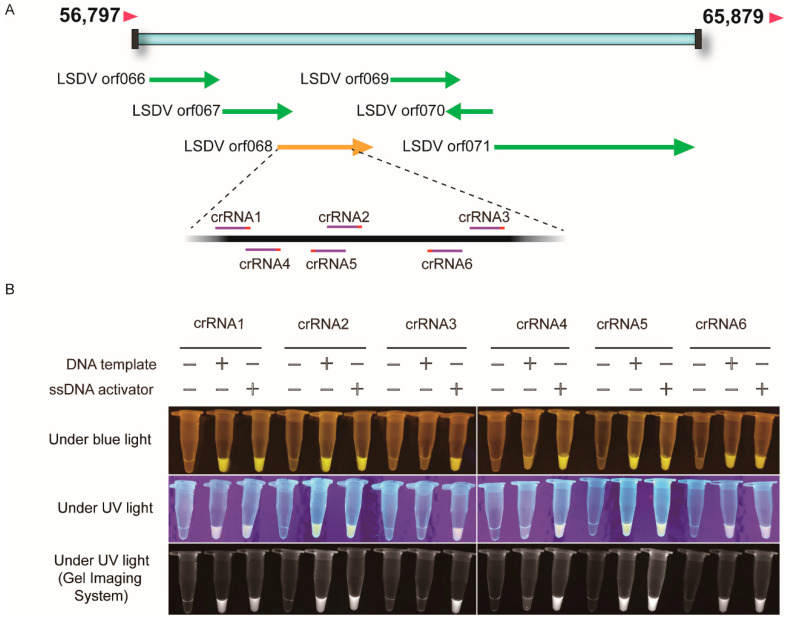
Identification of the highly active crRNAs for LSDV *orf068* gene using CRISPR-Cas12a enhanced fluorescence assay. (**A**). Schematic diagram of the position of crRNAs in LSDV *orf068* gene. (**B)**. Identification of the highly active crRNAs. crRNA1, 2, 3, 4, 5, 6 represent different crRNAs targeting to LSDV *orf068* gene. DNA template represents PCR products of *orf068* gene fragment. LSDV, lumpy skin disease virus; crRNA, CRISPR RNA; ssDNA activator is a crRNA-complementary ssDNA. Under blue or UV lights, the pictures were captured under blue (470 nM) and UV lights by a smartphone camera and gel imaging system.

To develop a strategy for sensitive detection of LSDV based on CRISPR-Cas12a fluorescence assay, it was necessary to screen highly active crRNA to detect the coding gene of LSDV. Six crRNAs targeting *orf068* were designed (Figure 1A). The PCR products of *orf068* and ssDNA activator were used as the templates to evaluate crRNA activity. As shown in Figure 1B, we found that all the Cas12a/crRNA complexes could cleave the ssDNA activator. Among them, crRNA1, crRNA2, crRNA5, and crRNA6 showed high activity on the dsDNA template and ssDNA activator under blue or UV light. Thus, crRNA1 was randomly selected as the active crRNA for subsequent experiments.

### 3.2. LSDV Detection Sensitivity by RPA-Cas12a-Fluorescence Assay

We first evaluated the LSDV detection sensitivity of PCR in combination with CRISPR-Cas12a. The plasmid containing the *orf068* gene was 10-fold serially diluted from 5 × 10^7^ to 5 × 10^−1^ copies/μL and used as the PCR amplification templates. We found that the sensitivity of PCR in combination with CRISPR-Cas12a was the same as the traditional PCR method, which was 5 × 10^4^ copies/μL (Figure S1A–C). To improve the LSDV detection sensitivity, we combined RPA with the CRISPR-Cas12a-based technique. Ten pairs of RPA primers were designed, and the RPA products amplified at 37 °C were subjected to 1% agarose gel electrophoresis (Figure S2). All the RPA products could be cleaved by Cas12a/crRNA1 complex (Figure S2B). The amplification efficiency of RPA was relatively high when primer pairs 3, 4, 7, and 9 were used (Figure S2A,B).

**Figure 2:**
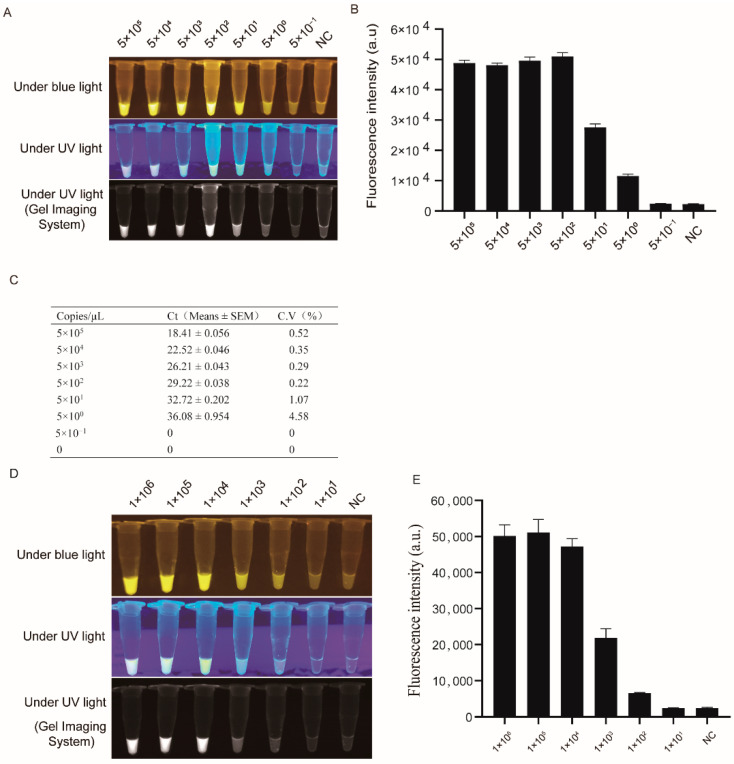
Comparison of the detection sensitivity between RPA-Cas12a-fluorescence assay and quantitative real-time PCR assay. (**A**). Fluorescent signals of different dilutions by RPA-Cas12a-fluorescence assay under blue or UV light. (**B**). The fluorescent intensity of the RPA-Cas12a-fluorescence assay was calculated by a multi-functional microplate reader. (**C**). The detection limit of different dilutions by quantitative real-time PCR. (**D**). Fluorescent signals from a series of 10-fold dilutions of LSDV titer (TCID_50_/mL) using the RPA-Cas12a-fluorescence assay. (**E**). The fluorescent intensity of the RPA-Cas12a-fluorescence assay for detection of a series of 10-fold dilutions of LSDV titer (TCID_50_/mL) was calculated by a multi-functional microplate reader. A series of 10-fold dilutions of pUC57-*orf068* plasmid (copies/μL) was calculated (**A**–**C**). In figures (**A**,**B**,**D**,**E**), RPA primer pair 4 (RPA-4F/R) was used. In figure C, *Orf068*-qPCR-F/R primer was used. NC, negative control; Ct, cycle threshold. SEM, standard error of the mean; C.V, coefficient of variation. Under blue or UV lights, the pictures were captured under blue (470 nM) and UV lights by a smartphone camera and gel imaging system.

Of these four RPA primer pairs, we found that the LSDV detection sensitivity of PRA was relatively high when primer pair 4 was combined with CRISPR-Cas12a (Figure 2A and Figure S3). Therefore, primer pair 4 was selected for subsequent RPA amplification. By using the same 10-fold serially diluted plasmid containing *orf068* as the amplification template, the limit of detection (LOD) of the RPA-Cas12a-fluorescence assay was improved to 5 copies/μL under blue light or UV light (Figure 2A). This LOD value was consistent with that detected by the fluorescence microplate reader (Figure 2B). In addition, the LSDV detection sensitivity by RPA-Cas12a-fluorescence assay was consistent with that of the traditional qPCR method (Figure 2C).

Furthermore, the LSDV detection sensitivity was evaluated with viral DNA as the template. The LSDV was 10-fold serially diluted from 1 × 10^6^ TCID_50_/mL to 1 × 10^1^ TCID_50_/mL. The LOD of the RPA-Cas12a-fluorescence assay for LSDV detection was 1 × 10^2^ TCID_50_/mL (Figure 2D,E). Afterward, the reaction time of the RPA-Cas12a-fluorescence assay was further optimized to 15 min for sensitive LSDV detection (Figure S4A–D).

### 3.3. LSDV Detection Specificity by RPA-Cas12a-Fluorescence Assay

**Figure 3:**
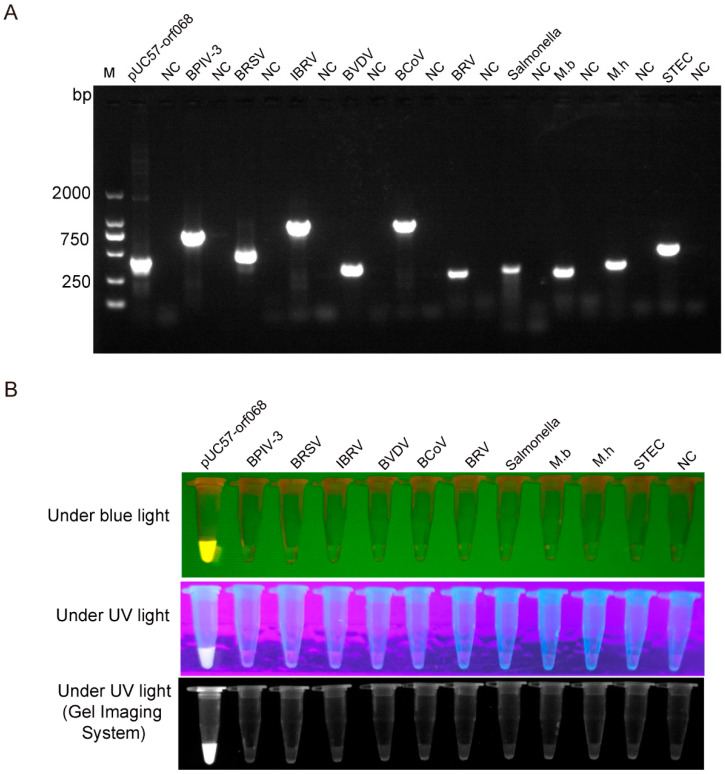
Specificity of the RPA-Cas12a-fluorescence assay for detection of the LSDV. (**A**). Agarose gel electrophoresis of PCR amplification products. (**B**). Specificity of the RPA-Cas12a-fluorescence assay for LSDV detection. Lane M, DNA ladder; Lane NC, negative control; pUC57-*orf068* used as a positive control; BPIV-3, Bovine parainfluenza virus type 3; BRSV, bovine respiratory syncytial virus; IBRV, Infectious Bovine Rhinotracheitis Virus; BVDV, Bovine Viral Diarrhea Virus; BCoV, Bovine coronavirus; BRV, Bovine Rotavirus; M.b, Mycoplasma bovis HB0801; M.h, A1 Mannheimia Haemolytica; STEC, Shiga toxin-producing Escherichia coli; Under blue or UV lights, the pictures were captured under blue (470 nM) and UV lights by a smartphone camera and gel imaging system.

The DNA or cDNA of common bovine pathogens including BPIV-3, BRSV, IBRV, BVDV, BCoV, BRV, *Salmonella enterica Dublin*, *M.b*, *M.h,* and *STEC* were used as templates to evaluate the specificity of RPA-Cas12a-fluorescence assay with pUC57-*orf068* as positive control and sterile ddH_2_O as a negative control. Firstly, the conventional PCR was used to amplify the target genes of all the pathogens and plasmid (Figure 3A), and then the PCR amplification products were subjected to Cas12a digestion to verify the pathogen. The primer pair 4 was used to perform RPA amplification, then the RPA amplification products were subjected to Cas12a digestion to test the specificity of the crRNA1 and the primer pair 4. The Cas12a assay results revealed that only the synthetic *orf068* gene generated a positive fluorescence signal, while all other DNA or cDNA were negative (Figure 3B).

To assess whether background bovine DNA interfered with LSDV detection by RPA-Cas12a-fluorescence assay, the RPA assay of LSDV *orf068* was performed in the presence or absence of 100 ng of background bovine DNA extracted from MDBK cells. Further, the products of the RPA reaction were digested by Cas12a. In the Cas12a assay, the synthetic *orf068* gene with or without cattle genomic DNA (gDNA) generated similar positive fluorescence signals under blue light and UV light (Figure S5A). The multi-functional microplate reading results showed that significantly higher fluorescence intensity was observed in *orf068* groups (with or without bovine DNA) than in the negative control group (with or without bovine DNA). These results indicated that background bovine DNA had no effect on LSDV detection by RPA-Cas12a-fluorescence assay (Figure S5B).

### 3.4. Detection of LSDV in Clinical Samples by RPA-Cas12a-Fluorescence Assay

**Figure 4:**
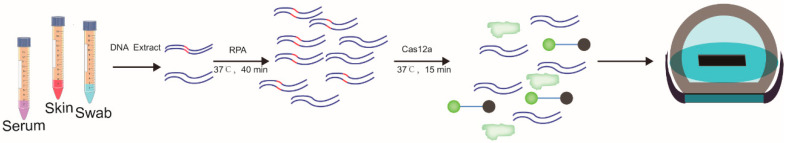
Schematic of the assay for rapid detection of the LSDV. RPA, recombinase polymerase amplification.

The results of the qPCR assay and RPA-Cas12a-fluorescence assay of the 40 clinical samples with known backgrounds were compared. As shown in Figure 4, the samples were tested with three replicates. The qPCR assay revealed 27 positive samples (out of 40 samples), similarly, the RPA-Cas12a-fluorescence assay detected 27 positive samples (out of 40 samples), with 26 positive samples overlapped in two assays and one positive sample which was detected as opposite in the two assays (Table 1). Therefore, the diagnostic sensitivity and specificity of the RPA-Cas12a-fluorescence assay were 96.3% (95% CI: 81.0–99.9%) and 92.31% (95% CI: 62.1–99.6%), respectively (Table 1), and our RPA-Cas12a-fluorescence assay results exhibited high similarity to that of conventional qPCR assay.

## 4. Discussion

The LSD is a newly emerging infectious disease causing serious economic losses in the Chinese cattle industry [6,32]. A simple, efficient, sensitive, and visual LSDV detection method is critical for the early prevention and control of the spread of LSD. This study is aimed to reduce the LSDV detection cost, time, labor, and requirements on equipment and technical eligibility. However, traditional detection methods either at the molecular level or serological level cannot meet these demands. Although RPA technology is fast and sensitive, with results generated within 1 h, the detected samples are extremely easily contaminated by aerosols. By overcoming the above-mentioned shortcomings, our established RPA-Cas12a-fluorescence assay can conveniently and sensitively detect the LSDV.

Our assay is more sensitive than conventional PCR recommended by OIE but less sensitive than qPCR. The sensitivity of our assay can reach the single-copy level for plasmid and about 10^2^ TCID_50_/mL for LSDV gDNA. The advantage of our RPA-Cas12a-fluorescence assay is its simple operation, which requires only a water bath or an incubator. Further, it takes only 15 min to complete one test, taking much less time than qPCR and PCR. Meanwhile, the fluorescent signal is visible with the naked eye under an inexpensive portable blue or UV light transilluminator. In addition, the results can be semi-quantified by using a microplate reader and qPCR system.

A primer is critical for the RPA reaction. We optimized the primers by avoiding poly-G and selecting C at the 5′ end, selecting G or C at the 3’ end, avoiding poly A, T, G, and C, and ensuring GC content within a range of 30% to 70%. Further, highly-conserved primer pairs were designed for the detection of wide-spectrum strains. The potential of the *orf068* gene as a detection target has been validated by qPCR methods [15,31]. Unfortunately, although we selected the conserved *orf068* gene and its conserved fragment as the target, the wide applicability of this gene cannot be validated because there is only one genotype of the LSDV in China. The activity of crRNA is critical for increasing the sensitivity of this assay. Six highly-scored crRNAs were designed within 300 bp in the *orf068* gene, of which, crRNA1 showed the highest cleavage activity. However, the satisfactory application of the Cas12a assay to other virus detection lends support to our LSDV RPA-Cas12a-fluorescence assay. The CRISPR-Cas12a combined with the RPA or LAMP assay has been applied to the detection of ASFV [18], SARS-CoV2 [22,23,25], mycoplasma [33], and *Mycobacterium tuberculosis* [26]. The LOD of the CRISPR-Cas12a + RPA/LAMP assay is <100 copies/µL or 10 aM, indicating that this assay is significantly more sensitive than PCR and comparable to qPCR. Furthermore, its specificity is superior to traditional PCR and qPCR because of the special primer design principle and unique crRNAs.

Notably, our data indicated that one sample was determined as positive by qPCR, but determined as negative by the RPA-Cas12a-fluorescence assay (Table 1). The possible reason for such a difference lies in the Cycle Threshold (Ct) value of this sample (35.34) representing a low virus loading, which was under the LOD of the RPA-Cas12a-fluorescence assay (Figure 2D,E). Another sample was determined as negative by qPCR, but as positive by the RPA-Cas12a-fluorescence assay. This difference might be attributed to the possibility that abundant RPA reactions within a short time resulted in the contamination of this sample by aerosols. The more sensitive the detection method is, the easier the sample is to be contaminated. Many measures could be taken to reduce contamination, such as adding mineral oil, maintaining adequate ventilation, and separating the polluted areas from clean areas. Integrating the RPA with a Cas12a reaction in a single reaction can minimize aerosol pollution [25,33,34,35].

In summary, a highly sensitive and specific method for LSDV *orf068* gene detection has been developed in this study. Compared to the conventional methods, our RPA-Cas12a-fluorescence assay is simple, fast, accurate, and suitable for on-site tests. Therefore, our assay has a promising application in LSDV field detection and LSD control.

## Figures and Tables

**Table 1:** Comparison of clinical sample test results between qPCR assay and RPA-Cas12a-fluorescence assay.

		qPCR Test		Total
		+	−	
RPA-Cas12a-fluorescence assay	+	26	1	27
	−	1	12	13
Total		27	13	40

“+” represents a positive sample. “−” indicates a negative sample. RPA, recombinase polymerase amplification; qPCR, quantitative PCR.

## Data Availability

Not applicable.

## Supplementary Materials

The following supporting information can be downloaded at: https://www.mdpi.com/article/10.3390/genes13050734/s1, Figure S1: Comparison of the detection limit of CRISPR-Cas12a with PCR amplification; Figure S2: The efficiencies of ten pairs of specific RPA primers analysis of RPA reaction; Figure S3: Comparison of the detection limit of different RPA primers coupled with CRISPR-Cas12a; Figure S4: Optimization of the reaction time for rapid detection of LSDV; Figure S5: Evaluation of anti-interference ability with the detection of LSDV-orf068 gene in RPA-Cas12a-fluorescence assay; Table S1: Oligo sequences used in this study; Table S2: Single-guide RNAs used in this study.
