# Supplementary material for: Sensitive and Specific Detection of Lumpy Skin Disease Virus in Cattle by CRISPR-Cas12a Fluorescent Assay Coupled with Recombinase Polymerase Amplification
Source: Genes (Basel). 2022 Apr 22;13(5):734. doi: 10.3390/genes13050734 (PMC9141258; doi:10.3390/genes13050734)
Supplement: Supplementary file 1 [file genes-13-00734-s001.zip › genes-1680181-supplementary.pdf]

Supporting Information

Sensitive and specific detection of lumpy skin disease virus in cattle by CRISPR-Cas12a fluorescent assay coupled with recombinase polymerase amplification

Chuanwen Jiang [1,2], Dagang Tao [2], Yuanchen Geng[1, 2], Hao Yang[1,2], Bingrong Xu[3], Yingyu Chen[1, 2, 4, 5], Changmin Hu[2, 4, 5], Huanchun Chen[1, 2, 4, 5], Shengsong Xie[2,3,4*], Aizhen Guo[1,2,4,5*]

[1]The State Key Laboratory of Agricultural Microbiology, Huazhong Agricultural University, Wuhan, 430070, China,
[2]College of Veterinary Medicine, Huazhong Agricultural University, Wuhan, 430070, China,
[3]Key Laboratory of Agricultural Animal Genetics, Breeding and Reproduction of Ministry of Education & Key Lab of Swine Genetics and Breeding of Ministry of Agriculture and Rural Affairs, College of Animal Science and Technology, Huazhong Agricultural University, Wuhan, 430070, China,
[4]Hubei Hongshan Laboratory, Wuhan, 430070, China,
[5]Hubei International Scientific and Technological Cooperation Base of Veterinary Epidemiology, International Research Center for Animal Disease, Ministry of Science and Technology of the People's Republic of China, Wuhan, 430070, China,
[6]Key Laboratory of Development of Veterinary Diagnostic Products, Ministry of China Agriculture and Rural Affairs, Wuhan, 430070, China.
*Correspondence: Aizhen Guo, College of Veterinary Medicine, Huazhong Agricultural University, Wuhan, 430070, China. Email: aizhen@mail.hzau.edu.cn; Tel: 00862787287115; Shengsong Xie, College of Veterinary Medicine, Huazhong Agricultural University, Wuhan, 430070, China. Email: ssxie@mail.hzau.edu.cn.

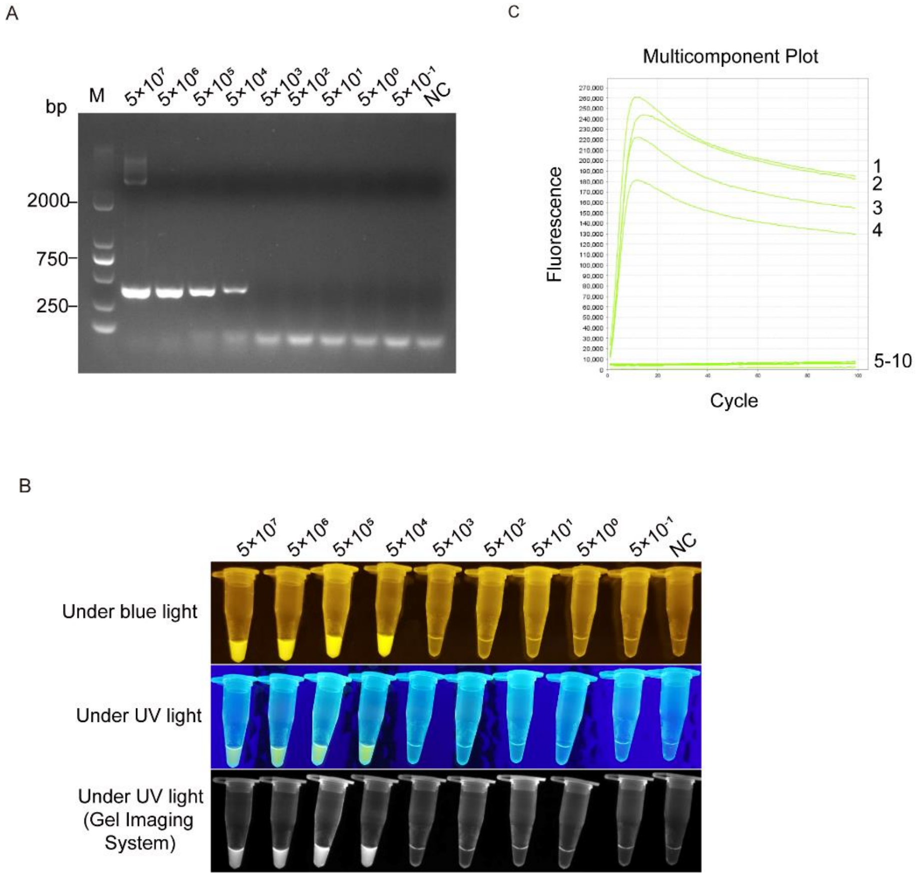

Figure S1. Comparison of the detection limit of CRISPR-Cas12a with PCR amplification. A. Products of the PCR reaction by using agarose gel electrophoresis analysis. Lane M, DNA ladder; bp, base pairs. **B**. Fluorescent signals from a series of 10-fold dilutions of pUC57-orf068 plasmid DNA (copies/μL) using the CRISPR-Cas12a wtih PCR amplification. **C**. Sensitivity of the CRISPR-Cas12a with PCR amplification detected by a real-time PCR system. Line 1, 5×10^7 copies/μL; line 2, 5×10^6 copies/μL; line 3, 5×10^5 copies/μL; line 4, 5×10^4 copies/μL; line 5, 5×10^3 copies/μL; line 6, 5×10^2 copies/μL; line 7, 5×10^1 copies/μL; line 8, 5×10^0 copies/μL; line 9, 5×10^{-1} copies/μL; line 10, negative control. NC, negative control; Under blue or UV lights, the pictures were captured under blue (470 nM) and UV lights by a smartphone camera and gel imaging system using UV light transilluminator.

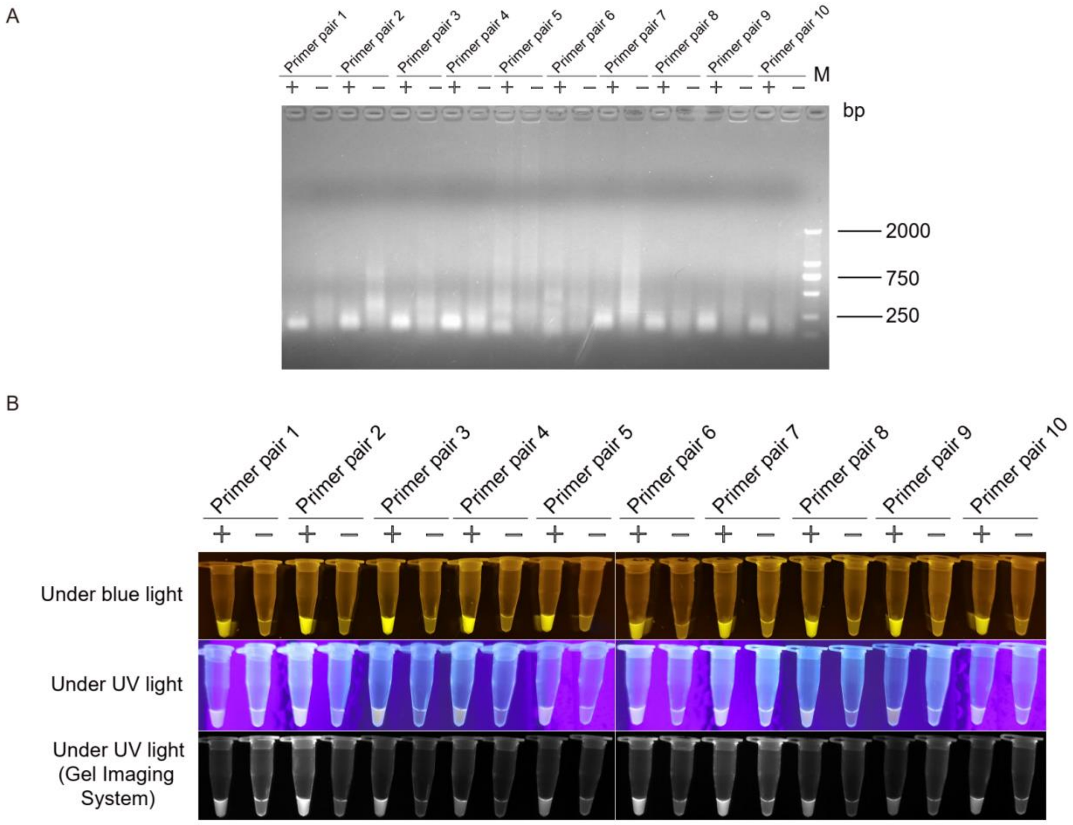

Figure S2. The efficiencies of ten pairs of specific RPA primers analysis of RPA reaction. A. The detection efficiencies of different primers by using electrophoresis analysis in RPA reaction. **B**. Fluorescence signals of LSDV *orf068* gene using RPA-Cas12a-fluorescence assay. Lane M, DNA ladder; bp, base pairs; Under blue or UV lights, the pictures were captured under blue (470 nM) and UV lights by a smartphone camera and gel imaging system using UV light transilluminator.

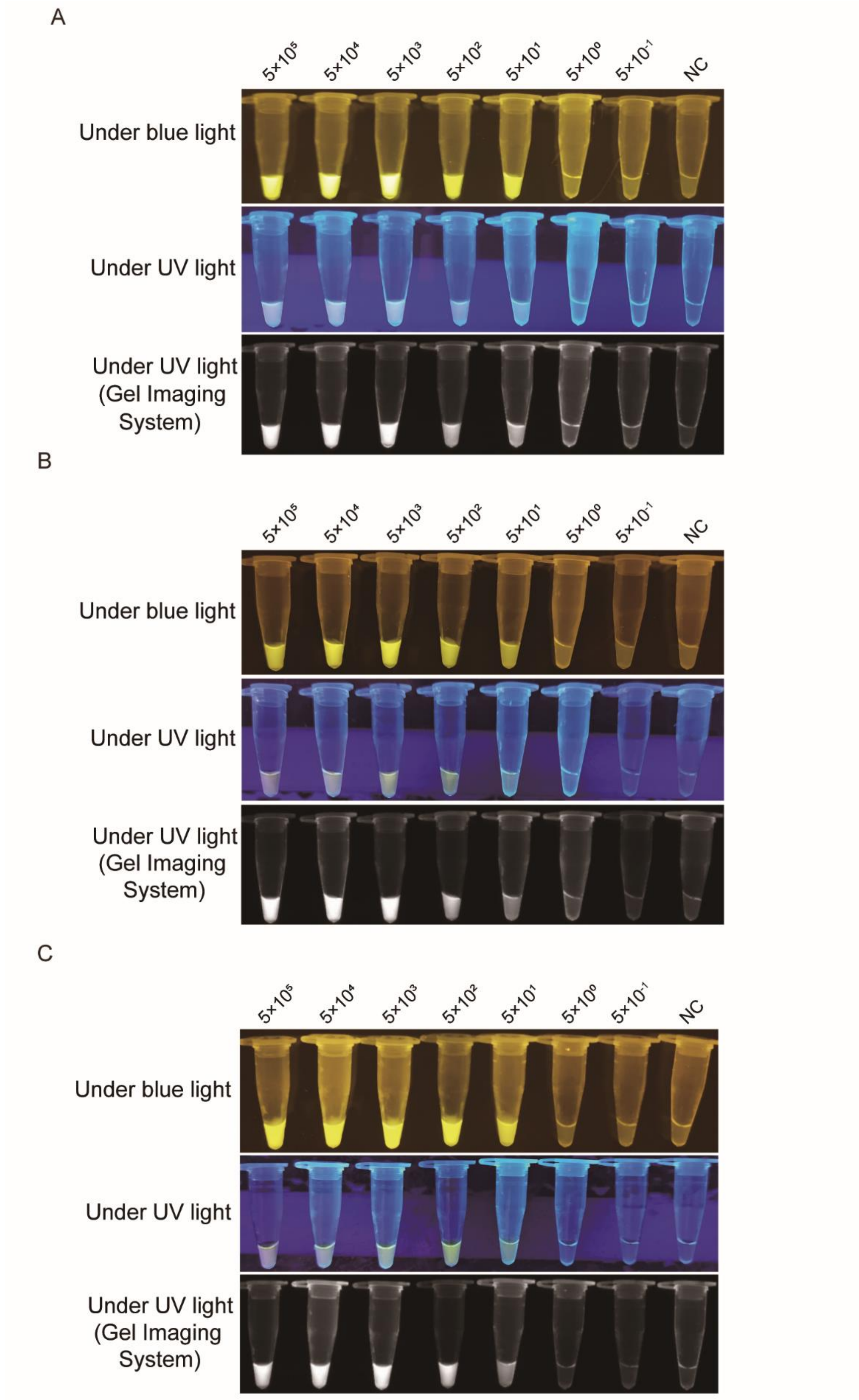

Figure S3. Comparison of the detection limit of different RPA primers coupled with CRISPR-Cas12a. A. Fluorescent signals from a series of 10-fold dilutions of pUC57-orf068 plasmid DNA (copies/μL) using the CRISPR-Cas12a

with third pair of RPA primer. **B**. Fluorescent signals from a series of 10-fold dilutions of pUC57-orf068 plasmid DNA (copies/μL) using the CRISPR-Cas12a based seventh pair of RPA primer. **C**. Fluorescent signals from a series of 10-fold dilutions of pUC57-orf068 plasmid DNA (copies/μL) using the CRISPR-Cas12a based ninth pair of RPA primer. NC, negative control; Under blue or UV lights, the pictures were captured under blue (470 nM) and UV lights by a smartphone camera and gel imaging system using UV light transilluminator.

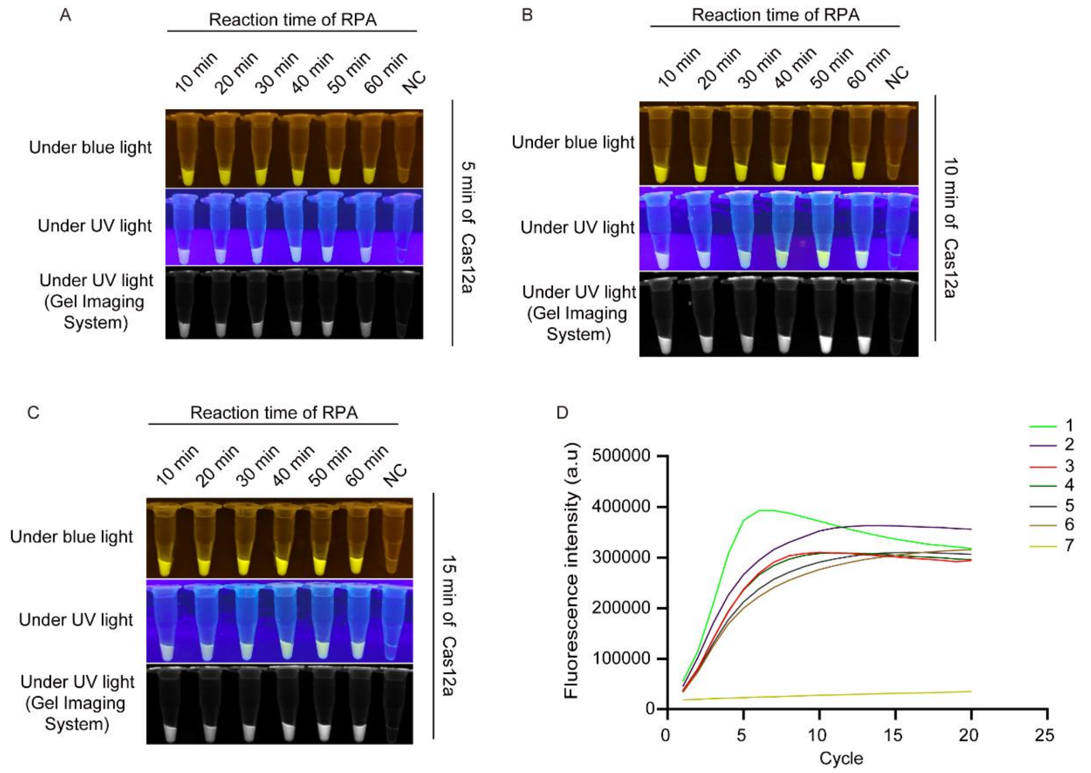

Figure S4. Optimization of the reaction time for rapid detection of LSDV. Fluorescence signals of Cas12a assay reacting 5 min (**A**), 10 min (**B**), 15 min (**C**) coupled with different RPA assay reaction time. **D**. Real-time fluorescence signal from CRISPR-Cas12a coupled with different RPA reaction time as monitored in a real-time PCR system. RPA, recombinase polymerase amplification; NC, negtive control; Lines 1-6 represent RPA reaction time: 60 min (1), 50 min (2), 40 min (3), 30 min (4), 20 min (5), 10 min (6); Line 7, 60 min (non-template control). Under blue or UV lights, the pictures were captured under blue (470 nM) and UV lights by a smartphone camera and gel imaging system.

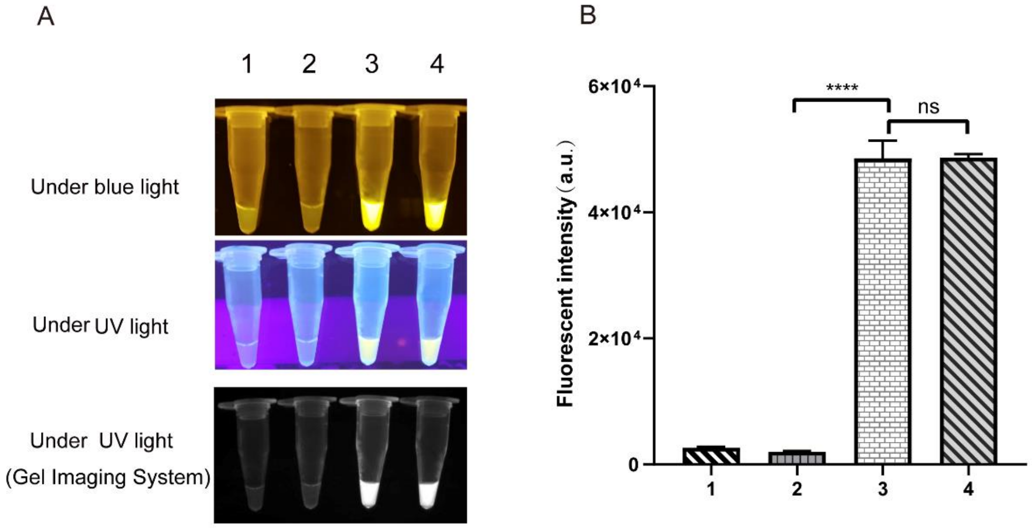

Figure S5. Evaluation of anti-interference ability with the detection of LSDV-orf068 gene in RPA-Cas12a-fluorescence assay. A. Fluorescent signals from PCR products of orf068 genes in cattle genomic DNA and pUC57-orf068 plasmid templates by RPA-Cas12a-fluorescence assay. **B**. Detection of pUC57-orf068 plasmid with or without cattle genomic DNA by the RPA-Cas12a-fluorescence assay using a multi-function microplate reader. Tube 1, DEPC treated water; Tube 2, cattle genome DNA; Tube 3, mixed pUC57-orf068 and cattle genome DNA; Tube 4, pUC57-orf068. Under blue or UV lights, the pictures were captured under blue (470 nM) and UV lights by a smartphone camera and gel imaging system. ****$p < 0.0001$; ns, no significant.

Supplementary Tables

Supplementary Table S1. Oligo sequences used in this study.

Application	Oligonucleotides Name	Sequences (5'-3')	Length of production (bp)
**sgRNA (*In Vitro* Synthesis)**	T7-crRNA-F	TAATACGACTCACTATAGG	
	Orf068-sgR1-R	TCATTTCTGCAGAATATTTAGGCGATCTACAACAGTAGAAAT	
	Orf068-sgR2-R	TTTTTAACATATTATACATGTGATATCTACAACAGTAGAAAT	
	Orf068-sgR3-R	TAAAACTTTTGATTTTATAGATGAATCTACAACAGTAGAAAT	
	Orf068-sgR4-R	AAAACTTGAAAATTGAATGGTGAAATCTACAACAGTAGAAAT	
	Orf068-sgR5-R	TGAAATGCTTCAACCATTTGCGCCATCTACAACAGTAGAAAT	
	Orf068-sgR6-R	TGAAAAAAAGATGTTTTATTTTAAATCTACAACAGTAGAAAT	
**PCR Primer**	LSDV-0rf068-F	GTCCATTCCCTGATCAATGGGT	401
	LSDV-orf068-R	ACGTTGTGTGGGTTCATAGTTTATT	
	BRSV-N-Outer primer-F	CAAACTAAATGACACTTTCAACAAG	
	BRSV-N-Outer primer-R	CATTTCATTCCTTAGTACATTGTTG	
	BRSV-N-Inner primer-F	CGTAGTACAGGTGACAACATTG	422
	BRSV-N-Inner primer-R	ACCAAAGCAGCAACACATAGCAC	
	BVDV-NADL-F	TCAGCGAAGGCCGAAAAGAGG	310
	BVDV-NADL-R	TCCATGTGCCATGTACAGCAGAG	
	BRV-VP6-F	CCACCAGGTATGAATTGGAC	231

	BRV-VP6-R	GAGTAATCACTCAGATGGCG	
	BPIV-N-F	GAGAAAGACCCAGGAAGAC AGA	704
	BPIV-N-R	ACACCCATCGCATAACTCCAG A	
	M.b-Uvrc-F	TAATTTAGAAGCTTTAAATGA GCGC	238
	M.b-Uvrc-R	CATATCTAGGTCAATTAAGGC TTTG	
	M.h-Hyp-F	CATTTCCTTAGGTTCAGC	306
	M.h-Hyp-R	CAAGTCATCGTAATGCCT	
	IBRV-TK-F	ACGGGCTGGGAAAGACAACA ACGG	868
	IBRV-TK-R	GCGGACACGTCCAGCACGAA CA	
	E.coli-pohA-F	GGTAACGTTTCTACCGCAGAG TTG	468
	E.coli-pohA-R	CAGGGTTGGTACACTGTCATT ACG	
	BCoV-N-F	TTGTAATTTTAATATGAGCAGC C	909
	BCoV-N-R	TTCTGCCAACTATTATAATAAG	
	Salmonella-stn-F	CTTTGGTCGTAAAATAAGGCG	260
	Salmonella-stn-R	TGCCCAAAGCAGAGAGATTC	
**qPCR Primer**	Orf068-qpcr-F	GGCGATGTCCATTCCCTG	68
	Orf068-qpcr-R	AGCATTTCATTTCCGTGAGGA	
**RPA primer**	RPA-3F	GGGTAAAAGATTTCTATATTCC TCACGGAAATG	131
	RPA-3R	ATCCTTTGTGATGCATCTAAGC TTTATAGGATT	
	RPA-4F	ATGGGTAAAAGATTTCTATATT CCTCACGGAAA	130
	RPA-4R	CTTTGTGATGCATCTAAGCTTT ATAGGATT	
	RPA-7F	GGTAAAAGATTTCTATATTCCT CACGGAAA	139
	RPA-7R	TATAGAATCATCCTTTGTGATG CATCTAAGC	
	RPA-9F	AATGGGTAAAAGATTTCTATAT TCCTCACG	133
	RPA-9R	TCCTTTGTGATGCATCTAAGCT TTATAGGATT	

**ssDNA-FQ reporters**	JOE-N12-BHQ1	/5′-JOE/GTATCCAGTGCG/3′BHQ1/	

Notes: crRNA, CRISPR RNA; ssDNA-FQ reporters, single-stranded DNA-fluorophore-quencher reporters; sgR, single guide RNA, bp, base pair.

Supplementary Table S2. Single guide RNAs (sgRNA) used in this study.

**sgRNAs ID**	**Sequences (5'-3')**	**PAM**
Orf068-sgR-1	CGCCTAAATATTCTGCAGAAATGA	TTTG
Orf068-sgR-2	ATCACATGTATAATATGTTAAAAA	TTTT
Orf068-sgR-3	TCATCTATAAAATCAAAAGTTTTA	TTTT
Orf068-sgR-4	TTCACCATTCAATTTTCAAGTTTT	TTTT
Orf068-sgR-5	GGCGCAAATGGTTGAAGCATTTCA	TTTA
Orf068-sgR-6	TTAAAATAAAACATCTTTTTTTCA	TTTA

Notes: PAM, protospacer adjacent motif.

Notes: The PCR amplification sequence of LSDV *orf068* gene is as follows:
>LSDV/Russia/Saratov/2017（Genbank: MH646674.1）(Partial vector sequences of pUC57-orf068, for PCR reaction)
TAATGGTAAGTATATTAAAACCAGCAGCATCTAGTTTAAAATGGCGATGTCCATTCCCTGATCAATGGGTAAAAGATTTCTATATTCCTCACGGAAATGAAATGCTTCAACCATTTGCGCCTAAATATTCTGCAGAAATGAGGTTAATAAGTATTTATAGCGGTAATCCTATAAAGCTTAGATGCATCACAAAGGATGATTCTATAAAATATGAAAAAAAGATGTTTTATTTTAATAAAATAATAAGGAATAGAATTATTATAAACTTTGATTATTCAAATCAAGAATATGACTTTTATCACATGTATAATATGTTAAAAACTGTATATTCTAATAAAGATTTTTCATCTATAAAATCAAAAGTTTTATTTTTTCACCATTCAATTTTCAAGTTTTTAAAAATACCTATATTCAACACAGAAAAAATAAACTATGAACCCACACAACGTAAAATACTTAGCAAAAATATTATGTTTAAAAACCGAAATATTAAAAAATCCATACGCTATAATAAGTAA
(The target sequence is highlighted in purple, the PAM sequence is highlighted in yellow)
